# Sleep, short-term memory, and mood states of volunteers with increasing altitude

**DOI:** 10.3389/fpsyt.2022.952399

**Published:** 2022-10-12

**Authors:** Huanhuan Wang, Xueyan Li, Jianhua Li, Yinghui Gao, Weihua Li, Xinke Zhao, Ruoqing Wen, Jiming Han, Kaibing Chen, Lin Liu

**Affiliations:** ^1^Peking University School of Nursing, Beijing, China; ^2^Gansu University of Chinese Medicine, Lanzhou, China; ^3^Department of Cardiology, The Second Medical Center & National Clinical Research Center for Geriatric Diseases, Chinese PLA General Hospital, Beijing, China; ^4^PKU-UPenn Sleep Center, Peking University International Hospital, Beijing, China; ^5^Gansu Armed Police Corps Hospital, Lanzhou, China; ^6^Sleep Center, The Affiliated Hospital of Gansu University of Chinese Medicine, Lanzhou, China; ^7^Medical College, Yan’an University, Yan’an, China; ^8^Department of Respiratory and Critical Care Medicine of the Second Medical Center & National Clinical Research Center for Geriatric Diseases, Chinese PLA General Hospital, Beijing, China

**Keywords:** short-term memory, mood states, volunteer, high altitude, sleep

## Abstract

**Purpose:**

This study sought to identify the changes and potential association between sleep characteristics and short-term memory, and mood states among volunteers at different altitudes and times.

**Method:**

A total of 26 healthy volunteers were recruited from the PLA General Hospital, and we conducted a longitudinal prospective survey for over 1 year from November 2019 to April 2021. First, we collected demographic data, sleep parameters by overnight polysomnography (PSG), short-term memory by digit span test, and mood states by completing a questionnaire with a brief profile of mood states among participants in the plain (53 m). Then, we continuously followed them up to collect data in the 3rd month at an altitude of 1,650 m (on the 3rd month of the 1-year survey period), the 3rd month at an altitude of 4,000 m (on the 6th month of the 1-year survey period), and the 9th month at an altitude of 4,000 m (on the 12th month of the 1-year survey period). Multiple linear regression analysis was used to construct models between sleep parameters and short-term memory, and mood states.

**Results:**

The prevalence of sleep apnea syndrome (SAS) significantly increased with rising elevation (*P* < 0.01). The apnea-hypopnea index (AHI), the mean apnea time (MAT), the longest apnea time (LAT), and the duration of time with SaO_2_ < 90% (TSA90) were increased (*P* < 0.05), and the mean pulse oxygen saturation (MSpO_2_), the lowest pulse oxygen saturation (LSpO_2_), and heart rate were significantly decreased with increasing altitude (*P* < 0.05). Digit span scores were decreased with increasing altitude (*P* < 0.001). A negative mood was more severe and a positive mood increasingly faded with rising elevation (*P* < 0.001). Additionally, linear correlation analysis showed that higher AHI, LAT, and MAT were strongly associated with a greater decline in short-term memory (in the 3rd and 9th month at an altitude of 4,000 m, respectively: *r*_s_ = −0.897, −0.901; *r*_s_ = −0.691, −0.749; *r*_s_ = −0.732, −0.794, *P* < 0.001), and also were strongly associated with more severe negative mood (in the 3rd month at altitudes of 1,650 m and 4,000 m, respectively: *r*_s_ = 0.655, 0.715, 0.724; *r*_s_ = 0.771, 0.638, 0.737, *P* < 0.000625). Multiple linear regression pointed out that AHI was a significant predictor of negative mood among people at different altitudes (in the 3rd month at an altitude of 1,650 m: TMD = 33.161 + 6.495*AHI; in the 3rd month at an altitude of 4,000 m: TMD = 74.247 + 1.589*AHI, *P* < 0.05).

**Conclusion:**

SAS developed easily in high altitudes, most often in CSA (central sleep apnea, CSA). The sleep, short-term memory, and negative mood were significantly more damaged with elevation in volunteers. Sleep parameters were closely associated with short-term memory and mood states in volunteers at high altitudes; the higher the sleep parameters (AHI, LAT, and MAT) scores, the more significant the mood disorders and the more obvious impairment of short-term memory. AHI was a critical predictor of the negative mood of volunteers at different altitudes. This study provides evidence that could help with the prevention and control of sleep disorder, cognitive disorder, and negative mood among populations with high altitudes.

## Introduction

Increasing numbers of people are traveling to high altitudes (HA), which are characterized by low atmospheric oxygen pressures that lead to long-term hypoxemia. Neurons in the brain are the most sensitive to hypoxemia. Thus, brain dysfunction is prone to develop at HA. Headache (1) and transient cerebral ischemia (2) were also common neurological symptoms associated with HA. And the reasons why individuals from low to high altitudes can develop sleep-disordered breathing (SDB) involved not only the stimulation of peripheral chemoreceptors of the carotid body which adjust the frequency and depth of breathing by detecting changes in arterial O_2_-7 and CO_2_-pH but also the instability of feedback control system resulting from the high gain in the system and changes in the ventilatory recruitment threshold (3, 4). In severe cases, people can develop mountain sickness, high-altitude cerebral edema, and high-altitude pulmonary edema (5, 6). Sleep disorders are common health problems for populations who reside at HA (7). Several studies reported a decrease in total sleep time, sleep fragmentation, and oxidative stress which were closely associated with neurocognitive decline and increased risk of developing Alzheimer’s disease (8–10). Additionally, the poor mood is a common problem among populations at an altitude above 900 m. Kious et al. (11) emphasized that anxiety and depression can easily occur and that the rates of suicidal ideation and suicide also increase among people who travel from low to high altitudes.

The importance of sleep is currently being recognized worldwide. In a report released by a mental health advisory body, it was highlighted that more than half of soldiers attributed their military mission failures to sleep deprivation (12). A large number of previous research has discussed how people sleep in high-altitude environments, first-time climbers of the plateau frequently feel tiredness, an increase in nightmares, sporadic awakenings, or dizziness upon waking, which reduces short-term memory, work capacity, and a propensity for errors (13). And Hansen et al. (14) discovered that individuals’ sympathetic impulses considerably increase in hypoxic conditions, keeping muscles moderately tense throughout sleep, increasing spontaneous micro-awakenings, and resulting in poor quality sleep; and Przybylowski et al. (15) found that when people traveled from high altitude to low altitude, their blood oxygen levels increased and their periodic breathing decreased during sleep. Liu’s team (16) discovered that individuals who spent a significant amount of time living at high elevations (>3,000 m) had a Pittsburgh Sleep Quality Index (PSQI) score of over 7 in 67.9% of all respondents, their most frequent complaints were about a longer sleep start and a shorter overall sleep duration. Li (17) and Liu et al. (18) investigated the effects of sleep disorders on soldiers’ military training performance and memory, and they discovered that the training performance (shooting performance) of soldiers who suffered from sleep disorders was considerably poorer than that of healthy control individuals. Reduced neurocognitive performance and a higher risk of developing Alzheimer’s disease were both significantly associated with decreased total sleep duration, sleep fragmentation, and reduced oxidative stress in persons. To summarize, we need to look after the health of sleep, cognitive function, and mood states in people living at high altitudes.

A review of previous studies showed that acute adjustment to high altitude can contribute to SDB, cognitive impairment, and poor mood. Patients with SDB are prone to anxiety and depression, which are closely related to the occurrence of cognitive impairment (7, 10, 11, 19), but the change and association between sleep parameters, short-term memory, and mood states with increasing altitude in volunteers are few literature reports. The current understanding of sleep at the plateau is mainly derived from subjects’ subjective answers, lacking objective evidence. This study sought to identify changes and potential relationships in sleep parameters, short-term memory, and mood states using PSG, digit span test, and BOMPS questionnaire by following volunteers at different altitudes, intending to serve as a reference for the creation of healthcare plans for maintaining human physical and mental health at high altitude.

## Methods

### Study population

A total of 26 healthy volunteers were recruited from the People’s Liberation Army General Hospital (PLA; Beijing, China) according to inclusion criteria as follows: (1) age ≥ 18 years; and (2) all volunteers were in good health without sleep, cognitive, or psychotic disorders. The exclusion criteria were as follows: (1) subjects living at a high altitude (>2,500 m); and (2) subjects suffering from physical, neurological, or mental disease. All participants were men, their mean age was 19.31 ± 1.26 y (minimum 18 y, maximum 22 y), and the body mass index (BMI) was 20.81 ± 2.08 kg/m^2^ (minimum 16.67 kg/m^2^ and maximum 25.69 kg/m^2^). Education was divided into three categories: 10 (38.5) volunteers performed 9 years of education means obtaining a junior high certificate, 12 (46.2) volunteers performed 12 years of education means obtaining a high school certificate, and 4 (15.4) volunteers performed 16 years of education means obtaining a university degree.

### Study design

**FIGURE 1:**
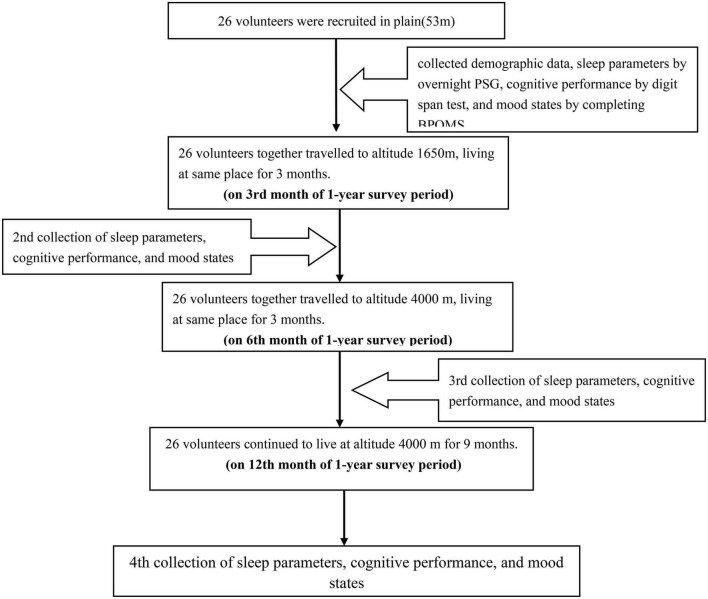
Technology road map. PSG, polysomnography; BPOMS, brief profile of mood states.

The study was a prospective, observational study, that started in November 2019 and ended in April 2021. First, we collected demographic data, sleep parameters by overnight polysomnography (PSG), short-term memory by digit span test, and mood states by completing a brief profile of mood states questionnaire (BPOMS) among participants recruited in the plain (53 m). Demographic data include sex, age, nationality, height, weight, BMI, and education. Because all volunteers had lived in the plains for a long time, they immediately traveled to the Mid-altitude (MA = 1,650 m) after completing data collection and safety notification. We recollected their data of sleep parameters, digit span score, and mood states score when all volunteers lived together at MA for 3 months (on the 3rd month of the 1-year survey period). Then, they continuously traveled to the high altitude (4,000 m), and data of volunteers were collected again when they lived in the same place for 3 months (on the 6th month of the 1-year survey period) and 9 months (on the 12th month of the 1-year survey period), respectively (Figure 1). According to the “International Diagnostic Criteria for Chronic High Altitude Sickness” formulated by the 6th International Conference on High Altitude Medicine in 2004, the plain was below 250 m, MA was between 250 and 2,500 m, and HA was above 2,500 m. This study was conducted in accordance with the Strengthening the Reporting of Observational Studies in Epidemiology (STROBE) reporting guidelines and the principles evinced in the Declaration of Helsinki. All subjects provided an informed consent form. The ethics committee of the PLA General Hospital approved the study (S2020-363-01).

### Polysomnography examination

Polysomnography (PSG) can exhibit the sleep characteristics of volunteers objectively. All participants have to undergo full overnight PSG (from 21:00 to 07:00 the next day) at various stages of this study. Their sleep parameters were recorded using a laboratory-based PSG instrument (Compumedics, Melbourne, Australia), including electroencephalography (EEG), electrooculography (EOG), electrocardiography (ECG), airflow measured by nasal pressure and oronasal thermistor, monitoring of respiratory effort with a chest and abdominal band, continuous pulse oximetry, body position, and snoring. Sleep apnea syndrome (SAS) was manually scored by PSG technologists according to the guideline of the American Academy of Sleep Medicine (AASM) (20) using the PSG data, and apnea was defined as the continuous cessation of airflow for more than 10 s. Hypopnea was defined as a 30% or greater drop inflow for 10 s or longer associated with ≥4% oxygen desaturation, and if either thoracic-abdominal breathing motions or snoring are present, the patient is considered to be suffering from obstructive hypopnea, while if neither is present, the patient is thought to have central hypopnea. AHI was the number of apnea and hypopnea episodes per hour during sleep, and SAS severity was defined according to AHI: mild SAS, 5 ≤ AHI < 15; moderate SAS, 15 ≤ AHI < 30; and severe SAS, AHI ≥ 30 (20). SAS generally refers to obstructive sleep apnea syndrome (OSA) and central sleep apnea syndrome (CSA). PSG recordings for one volunteer are presented in Supplementary Figure 1.

### Cognitive assessment

Cognitive assessment was based on the digital span test, which is mainly used to test short-term memory. The test consisted of two stages: digit span forward and backward tests, forward span captures attention efficiency and capacity, backward span is an executive task particularly dependent on working memory. Moreover, digit span assesses verbal/auditory memory (21). The software for the digit span test was developed according to the Wechsler adult intelligence scale (21). The test needed to be performed on IPAD. All subjects were arranged in a separate, quiet room to eliminate any sources of distraction. Before each test, instructions were presented on the screen to eliminate any variance introduced by a researcher explaining tests to participants. Then, a string of numbers would appear on the screen, and the participants needed to quickly remember and write the forward numbers on the screen (For digit span forward, if the instruction was “1 2 3,” the participant should repeat “1 2 3”). A string of numbers was composed of three Arabic numerals, four Arabic numerals, or five Arabic numerals, gradually increasing. Participants would get the corresponding score when a string of numbers was answered correctly (a string of numbers containing three Arabic numerals represented three points). Each string of numbers was assigned a score of 0 when answered incorrectly, and participants needed to answer a string of numbers with the same number of digits again and stopped if the answer is wrong again. The highest score was the final forward score of the participants. Immediately thereafter, the digit span backward test is started, in which the processing and scoring criteria are the same as those of the digit span forward test. However, participants needed to write the backward numbers on the screen (For digit span backward, if the instruction was “1 2 3,” the participant should repeat “3 2 1”). The total score of the digit span test equals the sum of the forward score and backward score, and higher scores represent the better cognitive performance of volunteers.

### Brief profile of mood states

The BPOMS questionnaire in this study, developed by McNair et al. (22) in 1992, was used to measure the mood state of the individual. This questionnaire consists of 40 adjectives designed to assess seven states (tension, anger, fatigue, depression, bewilderment, vigor, and self-esteem). Responses to each item were rated on a five-point Likert scale (0 indicates “Not at all” and 4 indicates “extremely”). The seven subscales of BPOMS can be combined into a total mood disturbance (TMD) score by summing the scores of the five negative mood subscales and subtracting the scores of the two positive mood states and adding a constant of 100. Cronbach’s α ranged from 0.726 to 0.888. Subjects answered the questionnaire individually.

### Statistical analysis

All data were analyzed using SPSS version 20.0 (SPSS Inc., Chicago, IL, United States). Metrological data were first tested for normality and homogeneity of variance. Normally distributed metrological data are expressed as the mean ± standard deviation (SD), and one-way analysis of variance was used for comparisons between groups. Metrological data that did not meet the criterion for normal distribution are expressed as the median (interquartile range), and non-parametric tests were used for comparisons between groups. Count data are expressed as the percentage (%), and chi-square and Fisher’s exact tests were used for comparisons between groups. Spearman’s correlation was utilized to analyze the relationships between sleep parameters and short-term memory, and the mood state of all participants at various stages. The Bonferroni correction was used for these multiple correlations. Based on spearman correlation analysis, confounding factors such as TST and HR were controlled. Short-term memory (forward, backward, and F+B scores) and mood states (tension, anger, fatigue, depression, bewilderment, vigor, and self-esteem), respectively, were the dependent variables and AHI, LAT, MAT, MSpO_2_, LSpO_2_, and TSA90 were the independent variables. Multiple linear regression analysis was performed. The difference was considered statistically significant when *P* < 0.05.

## Results

### Prevalence of sleep apnea syndrome at different altitudes

**FIGURE 2:**
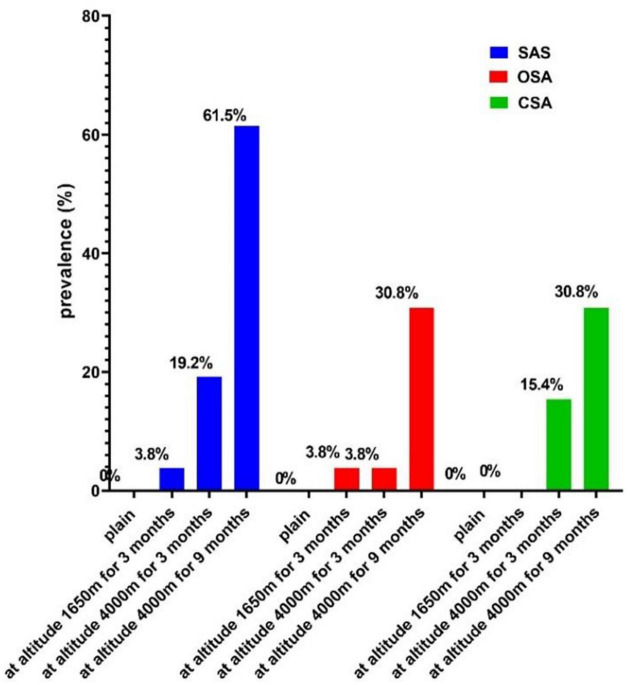
The prevalence of SAS at different altitudes. SAS, sleep apnea syndrome; OSA, obstructive sleep apnea; CSA, central sleep apnea.

No subjects developed SAS in the plain; 1 (3.8%) volunteer was clinically classified as having mild SAS when an altitude of 1,650 m was reached; 5 (19.2%) volunteers developed SAS at HA for 3 months, including 2 (7.7) volunteers who had mild SAS, 2 (7.7) volunteers had moderate SAS, and 1 (3.8) volunteer had severe SAS. A clinician classified 16 (61.5%) participants as having SAS at HA for 9 months, including 14 (53.8) volunteers with mild SAS, 1 (3.8) volunteer with moderate SAS, and 1 (3.8) volunteer with severe SAS. Therefore, the prevalence of SAS significantly increased with increasing altitude (*P* < 0.01). For these subjects, the prevalence of OSA and CSA also increased with rising elevation, which was both 30.8% on the 12th month of the 1-year survey period (*P* < 0.05; Figure 2).

### Short-term memory at different altitudes

**FIGURE 3:**
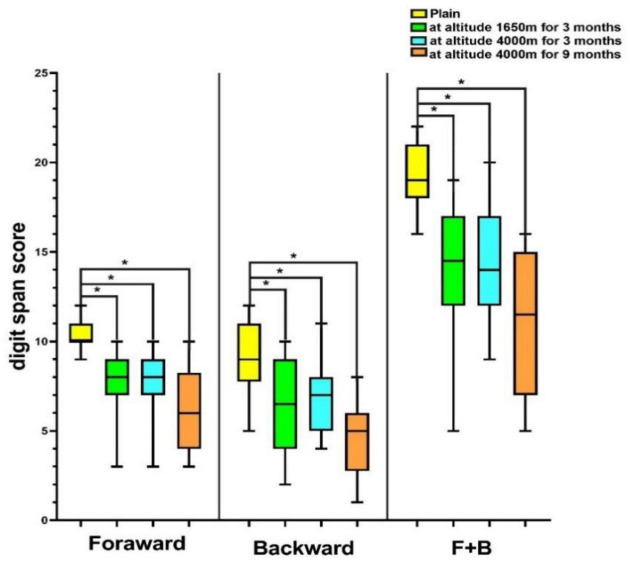
Digit span score of volunteers at different altitudes. *P < 0.05. F+B, forward+backward score.

In the plain, the mean digit span forward, backward, and forward+backward (F+B) scores were 10.19, 8.92, and 19.12, respectively. Of these, 15 (57.7%) of the respondents had backward scores above the mean (8.92), the forward scores of 19 (73.1%) volunteers, and F+B scores of 15 (57.7%) subjects were lower than the mean scores among subjects (10.19 and 19.12). In addition, 14 (53.5%), 13 (50%), and 13 (50%) volunteers’ forward, backward, and F+B scores were, respectively, higher than the mean value at the MA (7.69, 6.54, and 14.23). The forward, backward, and F+B scores of 15 (57.7%), 14 (53.5%), and 13 (50%) participants were higher than the mean values at HA for 3 months (7.69, 6.69, and 14.38). When HA was reached for 9 months, the backward and F+B scores of 17 (65.4%) and 14 (53.5%) volunteers were above the mean values (4.69, 11.00), and 12 (46.2%) forward scores were below the mean (6.31). Overall, the digit span scores of volunteers decreased with rising elevation (*P* < 0.001; Figure 3), and over half of the volunteers’ digit span scores were above the mean value at HA.

### Change in sleep parameters, short-term memory, and mood states with elevation

The sleep parameters, short-term memory, and mood states of all volunteers changed with rising elevation. The scores for sleep parameters including AHI, MAT, LAT, and TSA90 were increased (*P* < 0.05), but the MSpO_2_, LSpO_2_, and heart rates were significantly decreased with increasing altitude (*P* < 0.05). In terms of the digit span test, we found the downturn of the forward, backward, and F+B scores with increasing altitude (*P* < 0.05), but the digit span forward, backward, and F+B scores of volunteers reached HA for 3 months were slightly increased. The scores of negative mood including tension, anger, fatigue, depression, and bewilderment were elevated (*P* < 0.05), and positive mood scores including vigor and self-esteem were decreased with rising altitude (*P* < 0.05). However, the positive mood began to increase at HA at 3 months (Table 1).

**TABLE 1:** Characteristics of participants for different altitudes.

Indicators	Stage 1 (53 m)	Stage 2 (1,650 m)	Stage 3 (4,000 m)	Stage 4 (4,000 m)	*P*-value
**Sleep parameters**					
AHI*	0.00 (0.00, 0.10)^bc^	0.10 (0.00, 2.18)^d^	0.50 (0.00, 4.33)^f^	6.45 (2.58, 9.15)	<0.001
TST (min) *	455.46 ± 37.61	451.81 ± 39.29	446.75 ± 43.25	460.5 (439.65, 495.25)	0.387
LAT (s) *	0.00 (0.00, 4.50)^bc^	0.00 (0.00, 10.25)^de^	14.00 (0.00, 17.50)	21.00 (0.00, 57.75)	<0.001
MAT (s) *	0.00 (0.00, 3.25)^bc^	0.00 (0.00, 6.50)^de^	12.00 (0.00, 14.00)	16.50 (0.00, 21.08)	<0.001
MSpO_2_*	98.00 (95.00, 99.00)^abc^	94.00 (89.00, 97.00)^d^	92.00 (90.00, 92.25)	89.00 (86.00, 91.00)	<0.001
LSpO_2_*	90.42 ± 3.80^abc^	85.50 (79.75, 90.00)^d^	83.50 (81.00, 85.25)	80.00 (75.50, 82.00)	<0.001
TSA90 (min) *	0.00 (0.00, 13.00)^bc^	14.35 (0.00, 316.53)^d^	81.55 (13.23, 284.63)^f^	384.05 (208.30, 430.9)	<0.001
Heart rate**	63.46 ± 5.78^bc^	59.00 ± 6.58^d^	56.23 ± 5.32	53.81 ± 8.09	<0.001
**Mood state**					
Tension *	0.00 (0.00, 1.00)^bc^	1.00 (0.00, 3.00)^d^	2.50 (1.00, 6.00)	3.00 (1.00, 7.50)	<0.001
Anger*	0.00 (0.00, 0.00)^bc^	0.00 (0.00, 1.00)^de^	2.00 (0.00, 6.50)	2.00 (0.75, 6.50)	<0.001
Fatigue*	0.00 (0.00, 1.00)^bc^	1.00 (0.00, 3.00)	3.00 (0.75, 7.00)	4.00 (1.00, 7.25)	<0.001
Depression*	0.00 (0.00, 0.00)^bc^	0.00 (0.00, 1.00)^e^	1.00 (0.00, 3.00)	1.00 (0.00, 6.00)	<0.001
Bewilderment*	0.00 (0.00, 1.00)^bc^	1.00 (0.00, 3.00)	2.00 (0.75, 5.00)	2.00 (0.75, 5.25)	<0.001
Vigor*	23.00 (21.00, 24.00)^abc^	15.88 ± 6.45	13.85 ± 5.04	14.23 ± 4.85	<0.001
Self-esteem*	19.00 (16.00, 20.00)^abc^	9.35 ± 4.61	9.81 ± 4.28	10.19 ± 3.71	<0.001
NM*	0.00 (0.00, 3.00)^bc^	3.00 (1.00, 8.25)^e^	12.00 (3.75, 25.00)	13.00 (4.00, 34.00)	<0.001
PM*	40.50 (38.00, 43.00)^abc^	23.65 ± 8.86	27.00 (16.25, 30.25)	24.42 ± 8.22	<0.001
TMD*	60.50 (57.00, 65.25)^abc^	78.00 (71.75, 90.75)	87.50 (77.00, 103.50)	94.04 ± 21.08	<0.001
**Short-term memory**					
Forward score*	10.00 (10.00, 11.00)^abc^	8.00 (7.00, 9.00)	8.00 (6.00, 9.00)	6.31 ± 2.19	<0.001
Backward score*	8.92 ± 1.98^abc^	6.54 ± 2.32	6.69 ± 1.78^f^	5.00 (2.75, 6.00)	<0.001
F+B score*	19.00 (18.00, 21.00)^abc^	14.23 ± 3.22	14.38 ± 3.15 ^f^	11.50 (7.00, 15.00)	<0.001
SAS (%)***	0 (0)^c^	1 (3.8)^e^	5 (19.2)^f^	16 (61.5)	<0.001
OSA (%)***	0 (0)^c^	1 (3.8)^e^	1 (3.8)^f^	8 (30.8)	0.001
CSA (%)***	0 (0)^c^	0 (0)^e^	4 (15.4)^f^	8 (30.8)	<0.001

*Kruskal–Wallis test. **One-way ANOVA. ***Chi-squared test. Stage 1 compared to stage 2, ^a^P < 0.05; stage 1 compared to stage 3, ^b^P < 0.05; stage 1 compared to stage 4, ^c^P < 0.05; stage 2 compared to stage 3, ^d^P < 0.05; stage 2 compared to stage 4, ^e^P < 0.05; stage 3 compared to stage 4, ^f^P < 0.05. Stage 1: Plain; stage 2: at altitude 1,650 m for 3 months; stage 3: at altitude 4,000 m for 3 months; stage 4: at altitude 4,000 m for 9 months. AHI, apnea-hypopnea index; TST, total sleep time; LAT, the longest apnea time; MAT, the mean apnea time; MSpO_2_, the mean pulse oxygen saturation; LSpO_2_, the lowest pulse oxygen saturation; TSA90, the duration of time with Sao2 < 90%; NM, negative mood; PM, positive mood; TMD, total mood disturbance; F+B score, forward+backward score; SAS, sleep apnea; OSA, obstructive sleep apnea syndrome; CSA, central sleep apnea.

### The association between sleep parameters and short-term memory, mood states

Table 2 shows that the sleep parameters of subjects did not correlate with digit span score or mood states in the plain (*P* > 0.05). At MA, sleep parameters (AHI, LAT, MAT) of all volunteers had a strong positive relationship with negative mood (AHI: *r*_s_ = 0.684; LAT: *r*_s_ = 0.920; MAT: *r*_s_ = 0.615, *P* < 0.000625). When the volunteers reached the HA for 3 months, there was also a strong positive correlation between sleep parameters (AHI, LAT, MAT) and negative mood (AHI: *r*_s_ = 0.771; LAT: *r*_s_ = 0.638; MAT: *r*_s_ = 0.737, *P* < 0.000625), a strong negative relationship between sleep parameters (AHI, LAT, MAT) and digit span scores [(F+B scores) AHI: *r*_s_ = −0.897; LAT: *r*_s_ = −0.691; MAT: *r*_s_ = −0.732, *P* < 0.002]. At HA for 9 months, there was a positive correlation between AHI and negative mood (fatigue, depression) (*P* < 0.000625), and a strong negative relationship between sleep parameters (AHI, LAT, MAT) and digit span scores (F+B score) was still present (AHI: −0.901; LAT: *r*_s_ = −0.749; MAT: *r*_s_ = −0.794, *P* < 0.001; Table 2).

**TABLE 2:** The association between sleep parameters and short-term memory, and mood states derived from Spearman correlation analysis (*r*_s_).

		*r*_s_												
		Tension	Anger	Fatigue	Depression	Bewilder -ment	Vigor	Self- esteem	NM	PM	TMD	Forward score	Backward score	F+B score
**Stage 1**	AHI	–0.073	0.301	0.272	0.076	–0.028	–0.290	–0.361	0.086	–0.387	0.373	–0.014	0.048	0.087
	TST (h)	–0.283	–0.077	–0.339	–0.291	–0.314	0.145	0.071	–0.276	0.286	–0.439	0.083	0.265	0.350
	LAT	–0.145	0.285	0.211	0.049	–0.057	–0.241	–0.339	0.017	–0.330	0.289	–0.040	0.007	0.029
	MAT	–0.099	0.280	0.234	0.064	–0.041	–0.306	–0.370	0.052	–0.406	0.369	–0.019	0.070	0.107
	MSpO_2_	0.090	–0.213	–0.131	–0.293	–0.388	–0.156	0.120	–0.150	–0.025	–0.123	0.190	0.186	0.272
	LSpO_2_	0.134	–0.343	–0.073	–0.198	–0.357	0.125	0.232	–0.067	0.205	–0.299	0.188	0.313	0.404
	TSA90	–0.247	0.257	–0.048	0.034	0.156	0.062	–0.396	–0.029	–0.307	0.270	–0.482	0.031	–0.137
	Heart rate	–0.137	0.034	–0.039	0.122	–0.122	–0.057	0.234	–0.124	0.162	–0.193	–0.340	0.176	0.041
**Stage 2**	AHI	0.701*	0.609	0.528	0.668*	0.540	–0.614	–0.415	0.684*	–0.545	0.655*	–0.439	–0.272	–0.390
	TST (h)	0.207	–0.172	0.265	0.130	0.236	–0.035	0.055	0.218	0.053	0.060	0.283	0.157	0.255
	LAT	0.629	0.643*	0.432	0.701*	0.540	−0.666*	–0.537	0.596	−0.646*	0.715*	–0.350	–0.298	–0.378
	MAT	0.651*	0.640*	0.453	0.719*	0.549	−0.672*	–0.537	0.610	−0.644*	0.724*	–0.356	–0.261	–0.348
	MSpO_2_	–0.422	–0.319	–0.486	–0.589	–0.237	0.474	0.263	–0.368	0.416	–0.404	0.362	0.333	0.400
	LSpO_2_	–0.430	–0.476	–0.256	–0.343	–0.320	0.355	0.134	–0.440	0.262	–0.344	0.495	0.168	0.348
	TSA90	0.338	0.275	0.246	0.285	0.344	–0.423	–0.383	0.388	–0.447	0.469	–0.193	–0.421	–0.403
	Heart rate	–0.079	–0.371	0.109	0.006	–0.270	0.307	0.111	–0.161	0.212	–0.225	0.114	0.068	0.145
**Stage 3**	AHI	0.847*	0.878*	0.839*	0.875*	0.821*	–0.298	–0.240	0.920*	–0.294	0.771*	−0.850**	−0.777**	−0.879**
	TST (h)	0.032	–0.052	0.113	0.069	–0.013	0.058	0.038	0.025	0.126	0.009	0.190	0.204	0.255
	LAT	0.641*	0.715*	0.710*	0.640*	0.606	–0.324	–0.242	0.735*	–0.294	0.638*	−0.648**	−0.691**	−0.691**
	MAT	0.652*	0.732*	0.640*	0.767*	0.617	–0.418	–0.475	0.726*	–0.427	0.737*	−0.667**	−0.688**	−0.732**
	MSpO_2_	–0.270	–0.193	–0.138	–0.120	–0.310	–0.285	–0.134	–0.230	–0.233	–0.002	0.372	0.146	0.293
	LSpO_2_	–0.297	–0.460	–0.285	–0.396	–0.362	0.123	0.117	–0.363	0.174	–0.283	0.439	0.350	0.445
	TSA90	0.160	0.211	0.025	0.189	0.200	0.135	–0.102	0.157	–0.008	0.043	–0.437	–0.169	–0.335
	Heart rate	–0.245	–0.200	–0.294	–0.278	–0.153	0.074	0.099	–0.262	0.067	–0.273	0.378	0.396	0.443
**Stage 4**	AHI	0.337	0.613	0.679*	0.696*	0.546	–0.307	–0.117	0.615	–0.222	**0.557**	−0.878**	−0.836**	−0.901**
	TST (h)	–0.022	–0.010	–0.090	–0.107	0.034	0.093	0.137	–0.076	0.125	–0.074	0.007	0.057	0.044
	LAT	0.262	0.432	0.540	0.462	0.420	–0.155	–0.087	0.421	–0.140	0.388	−0.664**	−0.731**	−0.749**
	MAT	0.266	0.513	0.586	0.517	0.459	–0.176	–0.118	0.485	–0.169	0.453	−0.734**	−0.737**	−0.794**
	MSpO_2_	0.348	0.447	0.322	0.277	0.421	–0.291	–0.312	0.389	–0.344	0.487	–0.421	–0.410	–0.438
	LSpO_2_	0.232	0.455	0.294	0.336	0.291	–0.361	–0.362	0.292	–0.393	0.469	–0.404	–0.267	–0.317
	TSA90	–0.321	–0.492	–0.239	–0.253	–0.420	0.286	0.318	–0.350	0.310	–0.484	0.313	0.176	0.249
	Heart rate	–0.274	–0.310	–0.226	–0.345	–0.079	0.154	0.178	–0.309	0.167	–0.224	0.054	–0.055	–0.016

*The correlation p-value between sleep parameters and mood states (corrected with Bonferroni) was 0.05/80 = 0.000625. **The correlation p-value between sleep parameters and short-term memory (corrected with Bonferroni) was 0.05/24 = 0.002. Stage 1, plain; stage 2, in 3rd month at altitude 1650 m; stage 3, in 3rd month at altitude 4,000 m; stage 4, in the 9th month at altitude 4,000 m; AHI, apnea-hypopnea index; TST, total sleep time; LAT, the longest apnea time; MAT, the mean apnea time; MSpO_2_, the mean pulse oxygen saturation; LSpO_2_, the lowest pulse oxygen saturation; TSA90, the duration of time with Sao2 < 90%; NM, negative mood; PM, positive mood; TMD, total mood disturbance; F+B score, forward+backward score. The bolded values are the correlation coefficients, and the bold refer to P-values that are statistically significant.

### Sleep parameters were predictive of short-term memory and mood states

Short-term memory (forward, backward, and F+B scores) and mood states (tension, anger, fatigue, depression, bewilderment, vigor, and self-esteem) were used as dependent variables, and sleep parameters based on the above correlation analysis were used as independent variables (*P* < 0.05, Table 2) and confounding factors such as TST and HR were controlled. Diagnostic tests showed multicollinearity between LAT and MAT in MA; therefore, we removed an independent variable (MAT). Finally, we included the selected variables in the regression model. AHI was a significant predictor of negative mood at different altitudes (in the 3rd month at an altitude of 1,650 m: TMD = 33.161 + 6.495*AHI; in the 3rd month at an altitude of 4,000 m: TMD = 74.247 + 1.589*AHI, *P* < 0.05). The MSpO_2_ was a significant predictor of the F+B score in MA (F+B = −15.518 + 0.324* MSpO_2_, *P* < 0.01). AHI and MAT were significant predictors for short-term memory (F+B score) at HA for 9 months in the multiple linear regression model (F+B = −12.437 − 0.329*AHI − 0.150*MAT, *P* < 0.05, Table 3).

**TABLE 3:** Multiple linear regression analyses for the prediction of short-term memory and mood states from sleep parameters of volunteers (B).

		B												
		Tension	Anger	Fatigue	Depression	Bewilder -ment	Vigor	Self- esteem	NM	PM	TMD	Forward score	Backward score	F+B score
**Stage 2**	AHI	1.022**	–0.315	1.143**	0.993***	0.718**	–0.959	–1.063	4.231***	–2.312	6.495**	−0.482**	——	——
	LAT	–0.008	0.186**	–0.059	−0.078*	–0.003	–0.388	–0.116	0.145	–0.580	0.605	——	——	——
	MSpO_2_	–0.007	——	–0.086	–0.067	——	–0.032	——	——	–0.353	0.400	——	——	0.324**
	LSpO_2_	0.061	–0.029	——	——	——	——	——	0.292*	——	——	0.035	——	——
	SpO_2_<90	——	——	——	——	——	–0.007	——	——	–0.011	0.007	——	–0.005	–0.003
**Stage 3**	AHI	0.284***	0.412***	0.180*	0.222*	0.158**	——	——	1.248***	——	1.589***	−0.073*	–0.049	–0.110
	LAT	0.086	0.102	0.054	0.181	0.085	——	——	0.502	——	0.720	–0.018	–0.036	–0.049
	MAT	0.058	0.034	0.163	–0.057	0.039	–0.279	–0.149	0.266	–0.428	0.278	−0.118*	–0.088	–0.196
	LSpO_2_	——	–0.046	——	–0.013	——	——	——	——	——	——	–0.083	——	0.019
	SpO_2_<90	——	——	——	——	——	——	——	——	——	——	−0.005*	——	——
	HR	——	——	——	——	——	——	——	——	——	——	——	0.064	0.127
**Stage 4**	AHI	——	0.513*	**0.341**	0.361*	0.264*	——	——	1.702*	——	1.216	–0.159	−0.169*	−0.329**
	LAT	——	–0.083	–0.038	–0.038	–0.040	——	——	–0.174	——	——	0.004	0.028	0.032
	MAT	——	0.057	0.101	0.050	0.034	——	——	0.272	——	–0.096	–0.056	−0.094**	−0.150*
	MSpO_2_	——	0.028	——	——	0.131	——	——	——	——	1.523	0.008	0.032	0.032
	LSpO_2_	——	0.159	——	——	——	——	——	——	–0.343	0.165	–0.008	——	——
	SpO_2_<90	——	–0.006	——	——	–0.005	——	——	——	——	–0.018	——	——	——

*P < 0.05, **P < 0.01, ***P < 0.001; stage 2: in 3rd month at altitude 1,650 m; stage 3: in 3rd month at altitude 4,000 m; stage 4: in the 9th month at altitude 4,000 m; AHI, apnea-hypopnea index; TST, total sleep time; LAT, the longest apnea time; MAT, the mean apnea time; MSpO_2_, the mean pulse oxygen saturation; LSpO_2_, the lowest pulse oxygen saturation; TSA90, the duration of time with Sao2 < 90%; NM, negative mood; PM, positive mood; TMD, total mood disturbance; F+B score, forward+backward score. The bolded values are the B values, and the bold indicates that this indicator in the linear regression model is statistically significant.

## Discussion

In our study, all volunteers underwent a transition from low to mid-altitude followed by a transfer to high altitude, with at least 3 months of acclimatization at each stage, and the rising altitude was linked to an increase in the prevalence of SAS. The scores for sleep parameters including AHI, MAT, LAT, and TSA90 were increased, but the MSpO_2_, LSpO_2_, and heart rates were significantly decreased with increasing altitude. Meanwhile, volunteers showed impairment in short-term memory with elevation, but the digit span scores of subjects reached at HA for 3 months were slightly increased. Increased negativity and decreased positivity of volunteers were also observed. Multiple linear regression pointed out that higher AHI, LAT, and MAT scores were strongly associated with a greater decline in short-term memory at an altitude of 4,000 m, and were strongly associated with the more severe negative mood at altitudes 1,650 m and 4,000 m. These findings are important to help guide future intervention efforts for climbers reaching the top.

The prevalence of SAS (CSA and OSA) significantly increased with rising elevation in our study. Ortiz-Naretto et al. (23) reported that no mountaineers had periodic breathing (PB) at sea level, but that PB was frequent above 2,581 m and occurred in all subjects above 4,900 m. Liu et al. (24) demonstrated that newcomers who ascended to HA above 2,500 m often developed sleep-disorder breathing (SDB), manifesting as SAS, PB, and nocturnal hypoxemia, which can weaken already exhausted climbers. The above studies showed that acute exposure to high altitudes can damage sleep and breathing in healthy individuals. Not only that, in a study evaluating the effects of high altitude (2,761 m) on sleep apnea, Ju et al. (25) discovered that nocturnal oxygenation increased with time spent at altitude and that periodic breathing continued, and AHI increased as expected at HA but did not decrease over time. In a large, rigorously matched sample, the prevalence (77% vs. 54%, *P* < 0.001) and severity of SAS were considerably greater in highlanders than in lowlanders. Long-term residence at a high altitude (3,825 m) was also linked to lower SpO_2_ throughout wakefulness and sleep (26). Another study examining the influence of high altitude on sleep breathing events in children reported that students living at a high altitude (3,700 m) for a long time had considerably lower nocturnal MSpO_2_ (90.3 vs. 93.7 vs. 98.9, *P* < 0.05) and significantly greater DOI (8.1 vs. 3.1 vs. 0.7, *P* < 0.05) compared to individuals living at medium (2,500 m) and low altitude (500 m), and the more severe the sleep apnea, the higher the altitude (27), which is consistent with our findings. It is well-documented that PB, intermittent hypoxemia, and hypercapnia are typical characteristics of SAS (28, 29). Long-term hypoxia at high altitudes can induce enhancement in peripheral and central chemoreflexes, which has a complex interaction with cerebral blood flow, leading to higher loop gain and breathing instability, promoting the development of PB during sleep. Breathing was accelerated and deepened due to insufficient oxygen levels in the body, resulting in hyperventilation and hypocapnia, which stimulated the negative feedback system for controlling hypoxia and the subsequent inhibition of respiration, finally presenting as SAS. Respiratory pauses led to increased hypoxemia and consequent stimulation of ventilation and arousal as part of a vicious cycle (30). One explanation for the increase in obstructive events might be the cold factor and low relative humidity at high altitudes, which can cause damage to the mucous membranes of the upper respiratory tract of volunteers, resulting in nasal congestion and rhinitis, which can trigger or aggravate OSA. The nose is widely recognized for being the first part of the airway to come into touch with the external environment. When the nose is exposed to cold and dry air, the erectile tissue becomes congested and edematous to allow heat exchange and humidify the inhaled air, promoting an increase in nasal secretions and presenting symptoms of nasal congestion. Moreover, the loss of heat and water from the respiratory tract’s epithelial cells after prolonged exposure can result in dryness and crusting, predisposing them to rhinitis (31). Giraldo-Cadavid et al. (32) used PSG to evaluate children with rhinitis living at high altitudes and discovered that OSA occurred in 53% of the subjects and that a strong independent association emerged between the severity of rhinitis and the severity of the corresponding OSA (OR = 2.0, 95% CI: 1.12–6.04, *P* = 0.01). The above study tentatively confirms our suspicions, but more research is needed to probe the underlying mechanisms. Furthermore, the longer the volunteers stayed at high altitude (4000 m), the higher the prevalence of SAS became, the lower sleep SpO_2_ became in our study. A prospective study by Tellez et al. (28) found that the AHI of the population living at HA (3,800 m) for 12 months was higher than when they initially entered HA, but sleep SpO_2_ increased with time in HA, and AHI was always higher than the clinically considered serious level (AHI > 30) throughout the study. Ju et al. (25) also pointed out that AHI increased, and the night MSpO_2_ progress increased with the prolongation of time at HA. Subjects at the same HA (5,500 m) showed no diminution in the severity of CSA over 1 month (29). There are some differences with our study results, which may be due to the fact that the study population we included are young men who have a strong body metabolism and a high demand for oxygen, and it would take longer to adapt to hypoxia at HA. Volunteers’ sleep monitoring at HA lasted only 9 months, including two examinations that could not fully show the volunteers’ sleep at HA.

The digit span test is a method of testing short-term memory (one of cognitive function); we found the downturn of the digit span scores with increasing altitude, but the scores at HA for 3 months were slightly increased, which was consistent with Zhang et al.’s (33) study results that the values of digit span in subjects native to HA of 3,700 m, 4,500 m, and 5,100 m were significantly decreased when compared with subjects at sea level (forward score: 11.71, 10.26, 10.97 vs. 12.22; backward score: 6.89, 2.54, 6.48 vs. 8.37). Previous research has found that older adults who live at high altitudes for an extended period of time are more likely to develop cognitive impairment than those who live at low altitudes, with a prevalence of 94.7% (95 CI: 91.6–97.7%), which can easily progress to severe neurological disorders (dementia) over time, resulting in impaired functioning and lower quality of life (34). An investigation of acute and chronic exposure to high altitude on executive function, speed of processing, and memory in healthy children discovered that short-term 24-h exposure to high altitude significantly impaired short-term memory, situational memory, and executive function in healthy populations, with similar or even more severe impairments in these functions detected in children who had lived at high altitude for at least 3 years (35). Aside from the foregoing, it was also discovered that only long-term high altitude exposure impacted speed processing capacities. Oxygen is critical for neuronal functions and the growth of the brain. The higher the altitude, the lower the oxygen concentration in the air, and the short-term memory was more likely to be damaged. Shi et al. (36) also showed that rapid ascent to 4,280 m and remaining at this altitude for 3 h resulted in decreased audiovisual memory and short-term memory in all participants, suggesting that continuous plateau hypoxia can induce an obvious decrease in cognitive brain functions. However, Regard et al. (37) proposed that rapid ascent to high altitude had small effects on cognitive performance, subjects who developed acute mountain sickness at high altitude were mildly impaired in short-term memory, while subjects who remained healthy had a better short-term memory performance. At present, there is little data on how chronic exposure to high altitude affects cognitive function. Based on the results of cognitive function tests carried out by our team after 3 months of acclimatization at medium and high altitudes, the cognitive changes associated with hypoxia at high altitudes are still present, but more research is needed into the underlying pathophysiological causes.

We also observed significant changes in the sleep parameters and mood states among volunteers at different altitudes. AHI, MAT, LAT, TSA90, and negative mood scores were increased and MspO_2_, LspO_2_, HR, and positive mood were decreased with rising elevation. It is well known that SAS is strongly associated with AHI and SpO_2_ and that a hypoxic environment at HA can cause insufficient oxygen concentrations in individuals, affecting respiratory patterns during sleep either directly or indirectly, promoting SAS and hypoventilation (25). Several studies showed that the SaO_2_ of people with normal AHI decreased with rising elevation and that the MSaO_2_ during sleep was 97.3%, 87.0%, 83.0%, 71.0%, and 59.0% at 500, 2,640, 4,200, 6,400, and 8,400 m, respectively (38). Mood state is an effective index that reflects an individual’s mental health. Heinrich et al. (39) reported significantly higher rates of daytime fatigue and confusion in individuals at an altitude of 3,800 m compared to sea level, and a high correlation between mood disorders and altitude was also observed. Li et al. (40) demonstrated that mood states, such as stress, weariness, and vitality, worsen at altitudes above 6,000 m and increase with exposure time. Other studies have shown that acute exposure from low to high altitudes (>3,500 m) increases negative mood in individuals (41). This might be due to the simple fact that intermittent hypoxemia in HA caused the compensative hypoventilation of individuals, initiating hypocapnia, which itself may produce anxiety, low mood, and lead to hyperventilation (42). All the studies mentioned earlier have been conducted from the perspective of acute exposure plateaus, and studies on the effects of chronic exposure on human emotional states are uncommon, and the underlying mechanism was unknown. Moreover, the observation indexes of psychological states were mainly anxiety and depression, although fatigue was also a widespread problem for people living at HA in the present study.

Regression analysis showed that the sleep parameters of volunteers at HA were linked to mood states and short-term memory. The higher the AHI, LAT, and MAT were, the more severe the negative mood, and AHI was a significant predictor of negative mood among people who had reached HA. de Aquino Lemos et al. (30) reported an increase in depression, anger, and fatigue under hypoxic conditions of HA and positive correlations between sleep and mood states, including AHI with tension and arousal with mental confusion. A negative correlation between the efficiency of sleep and depression was also observed. Bian et al. (43) showed that SDB developed at HA was positively correlated with anxiety, and the synthesis of amines, substance P, catecholamines, and nitric oxide was affected due to breathing disruption during sleep, which is related to mood disturbances, anxiety, and stress. Reviewing previous literature, reactions, attention, and mood are significantly impaired during the first 1 to 2 weeks after an individual ascends to a high altitude. However, our study found that individuals who migrate to high altitudes for extended periods of time, after several months of acclimatization, do not reach the same mood states as at lower altitudes, considering that sleep disorders at high altitudes are closely related to mood disturbances. Asarnow et al. (44) demonstrated that sleep problems can predict the development of mood disorders. When sleep disorders or sleep deprivation occur, the amygdala in the brain, which is a key brain region for emotion, is abnormally reactive, increasing the level of response by almost 60%, thus affecting the individual’s mood state. Additionally, the higher the AHI, LAT, and MAT, the more apparent was the decline in short-term memory in our study. By tracing various indexes of subjects in different phases, we found that MSpO_2_ and MAT can predict short-term memory. Respiratory alterations during sleep were increased by hypoxia at HA, which caused neurodegenerative changes in the cerebral region and neurotransmitter systems that are involved in learning, memory, attention, and locomotive activity. Furthermore, under hypoxic conditions, recurrent respiratory interruptions at night might have increased oxidative stress and inflammation and decreased the cellular substrates and molecules of synaptic plasticity (30, 45). Studies by de Aquino Lemos et al. (30) and Frost et al. (45) support these findings. Participants who traveled from low altitude to HA of about 3,800 m showed that their short-term memory did not improve over time. Poor sleep quality and SDB contributed to impaired sustained attention and reaction times of subjects at HA. Evidence shows that SAS at HA was highly associated with cognitive performance and mood state. PSG is the gold standard for the diagnosis of SAS. Our study revealed that AHI, MSpO_2_, and MAT were good predictors of cognitive performance and mood states at different altitudes, which reflect the severity of SAS and the effect of treatment. Increasing AHI was closely associated with the prevalence of SAS including central and obstructive hypopnea events, which were associated with autonomic dysregulation, endothelial dysfunction, cardiac remodeling, and predisposition to cardiovascular diseases (30).

Longitudinal studies of the same population have highlighted the dynamic changes in sleep, short-term memory, and mood states with increasing altitude. This finding has direct implications for developing the characteristics of these observation indicators. However, our study population was little and only men, since the women were reluctant to participate in this study, which cannot represent the sleep characteristics, short-term memory, and mood states of a large sample of people at different altitudes. And future studies should have a longer follow-up period with repeated measurements on the same individual, which are easily prone to effects related to practice or fatigue. Furthermore, the research tools used may not be novel due to the long research time.

## Conclusion

Sleep apnea syndrome developed easily at HA. Sleep parameters, short-term memory, and mood states of volunteers were significantly altered with increasing altitude: sleep disorders were more severe, short-term memory was significantly impaired, and the mood state was markedly depressed. Sleep parameters were closely associated with short-term memory and mood states. AHI was a critical predictor of the negative mood of volunteers at different altitudes.

## Data availability statement

The raw data supporting the conclusions of this article will be made available by the authors, without undue reservation.

## Ethics statement

The studies involving human participants were reviewed and approved by the Ethics Committee of Chinese PLA General Hospital (S2020-363-01). The patients/participants provided their written informed consent to participate in this study. Written informed consent was obtained from the individual(s) for the publication of any potentially identifiable images or data included in this article.

## Author contributions

HW, XL, JL, YG, WL, XZ, and RW collected the data. HW and XL analyzed the data and wrote the manuscript draft. LL, JH, and KC designed this study. All authors contributed to the article and approved the submitted version.

## Abbreviations

stage 1: plain
stage 2: in the 3rd month at an altitude of 1,650 m
stage 3: in the 3rd month at an altitude of 4,000 m
stage 4: in the 9th month at an altitude of 4,000 m
AHI: apnea-hypopnea index
TST: total sleep time
TSA90: the duration of time with SaO_2_ < 90%
LAT: the longest apnea time
MAT: the mean apnea time
MSpO_2_: the mean pulse oxygen saturation
LSpO_2_: the lowest pulse oxygen saturation
SpO_2_: pulse oxygen saturation
NM: negative mood
PM: positive mood
TMD: total mood disturbance
F+B score: forward+backward score
SAS: sleep apnea
OSA: obstructive sleep apnea syndrome
CSA: central sleep apnea.
