# Supplementary material for: Sleep, short-term memory, and mood states of volunteers with increasing altitude
Source: Front Psychiatry. 2022 Oct 12;13:952399. doi: 10.3389/fpsyt.2022.952399 (PMC9600328; doi:10.3389/fpsyt.2022.952399)
Supplement: Supplementary file 1 [file Data_Sheet_1.pdf]

The PSG record of one of the volunteers we followed is shown in **Figure S-1.**

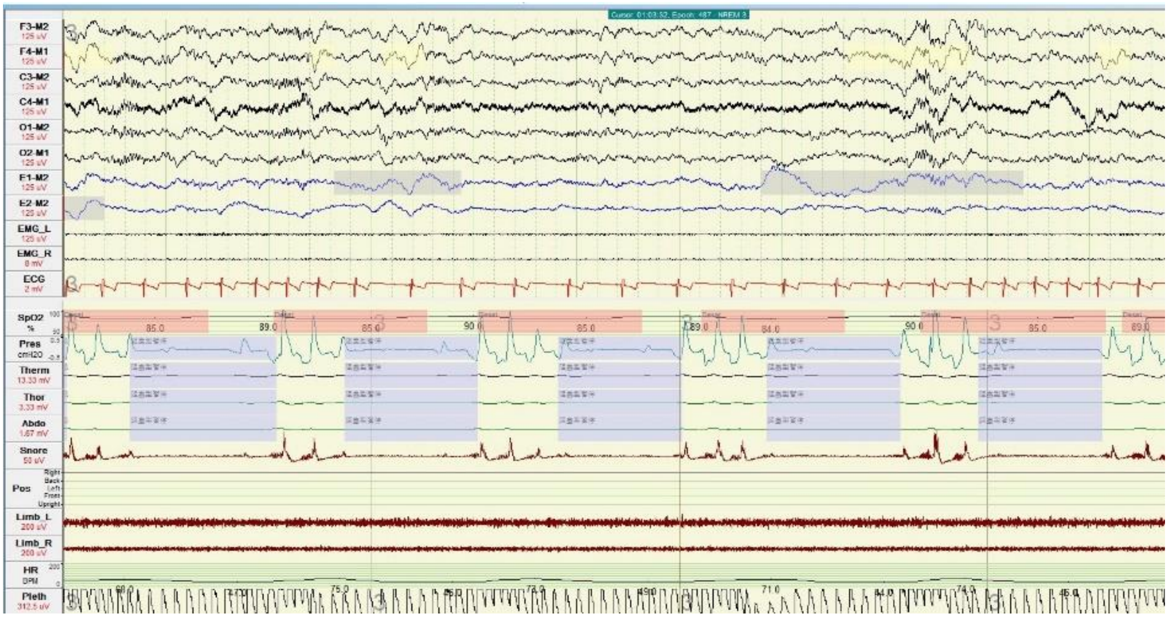

Figure S-1A: The PSG of a volunteer living at an altitude of 4,000 meters for 12 months

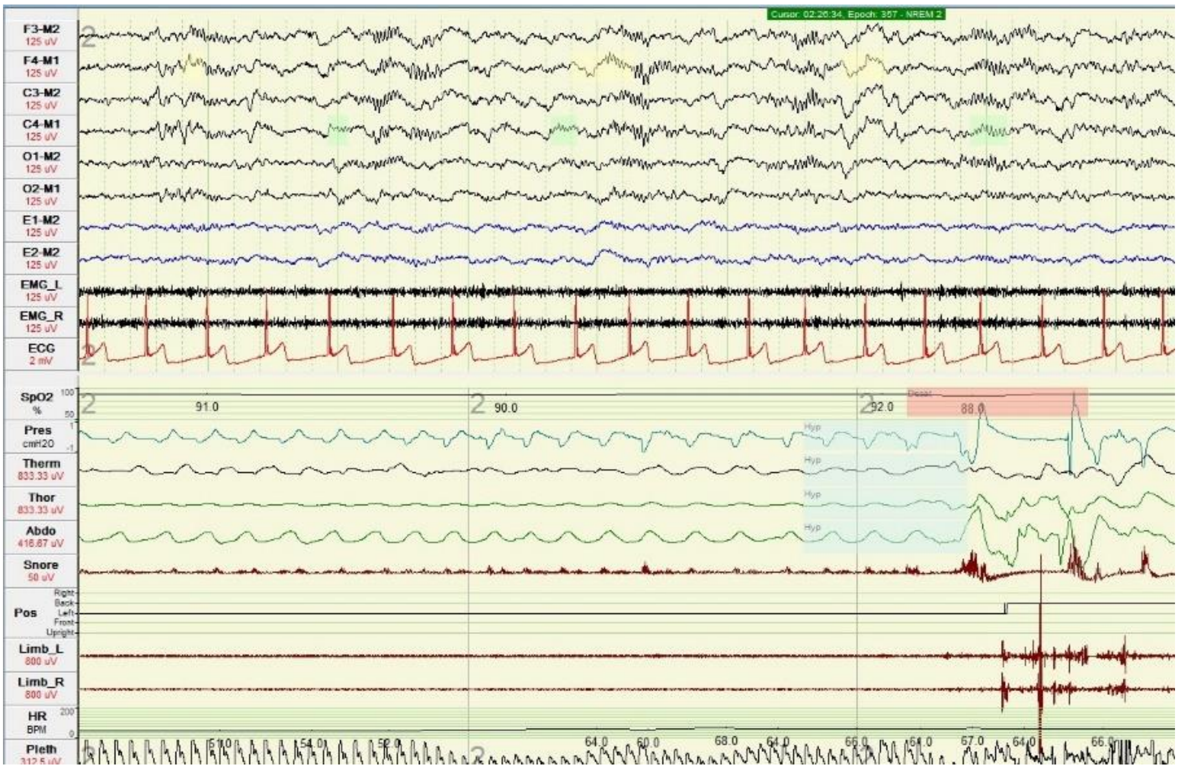

Figure S-1B: The PSG of a volunteer living at an altitude of 1650 meters for3 months

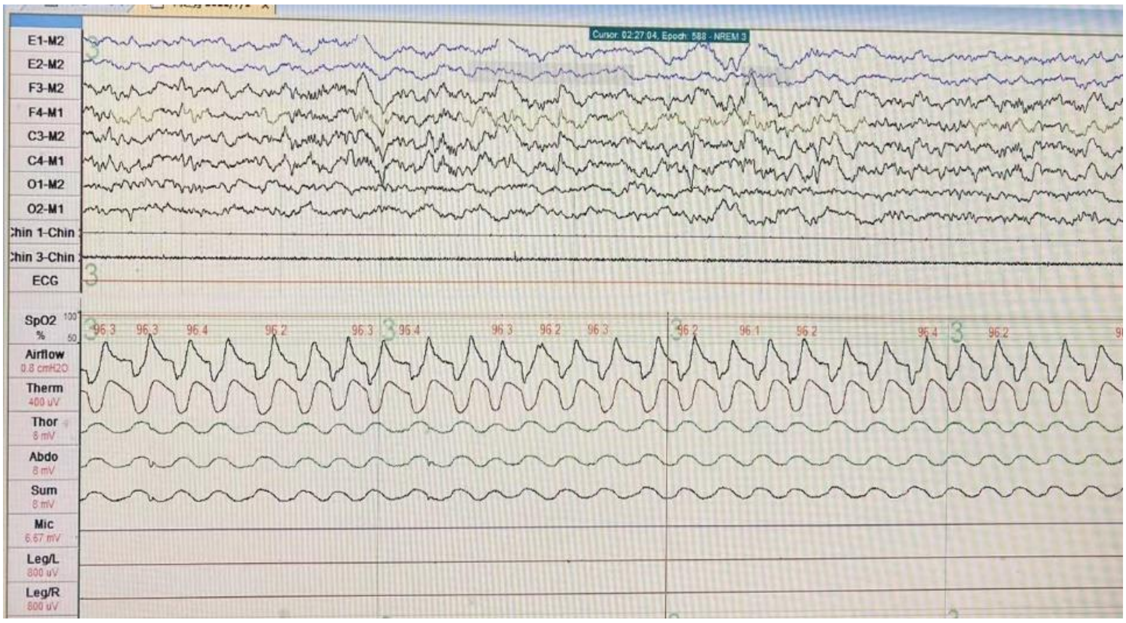

Figure S-1C: The PSG of a volunteer living at an altitude of 53 m
