# Supplementary figures and images for: Sleep, short-term memory, and mood states of volunteers with increasing altitude
Source: Front Psychiatry. 2022 Oct 12;13:952399. doi: 10.3389/fpsyt.2022.952399 (PMC9600328; doi:10.3389/fpsyt.2022.952399)

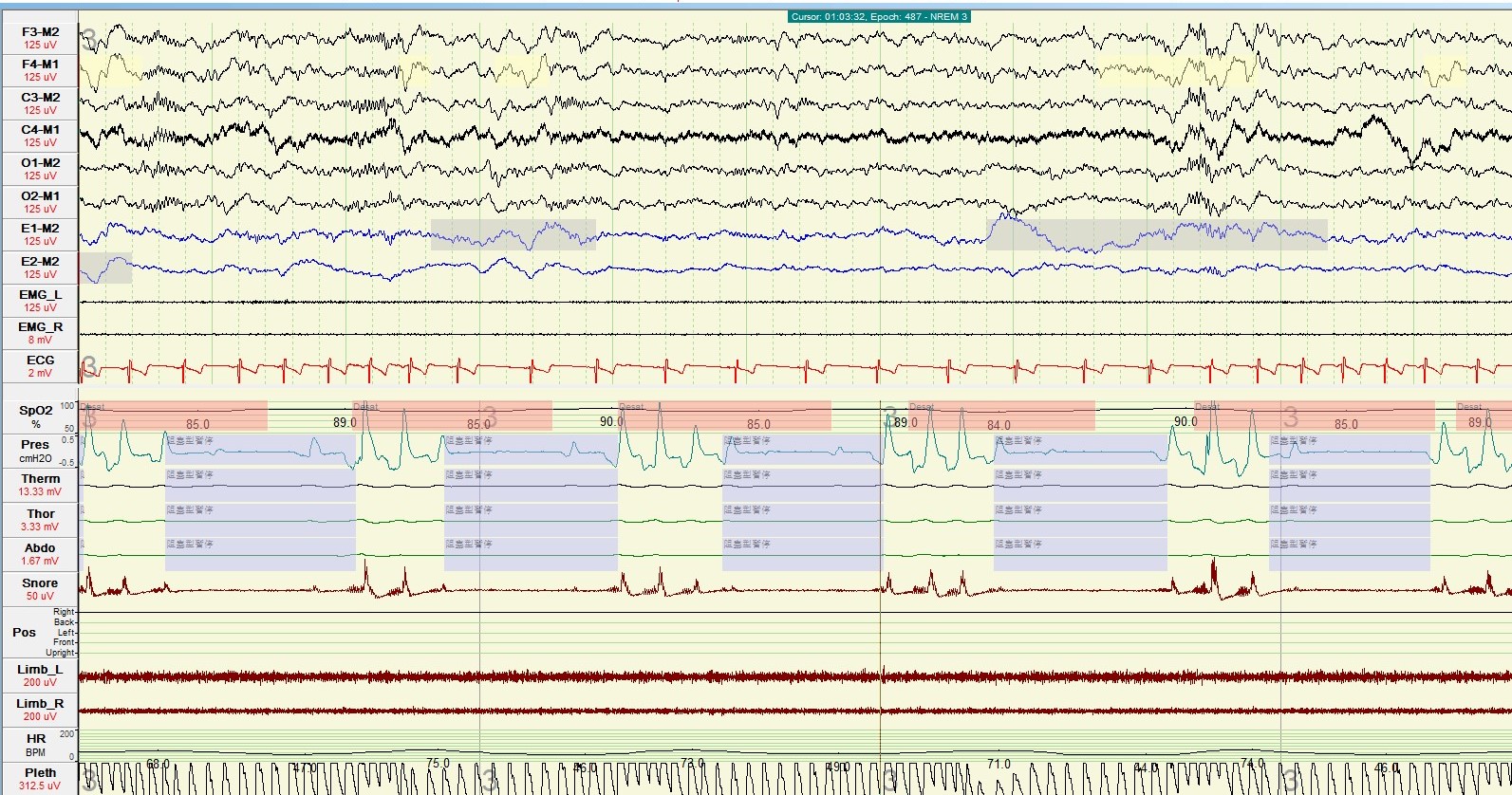

Supplement: Supplementary file 2 [file Image_1.jpg]

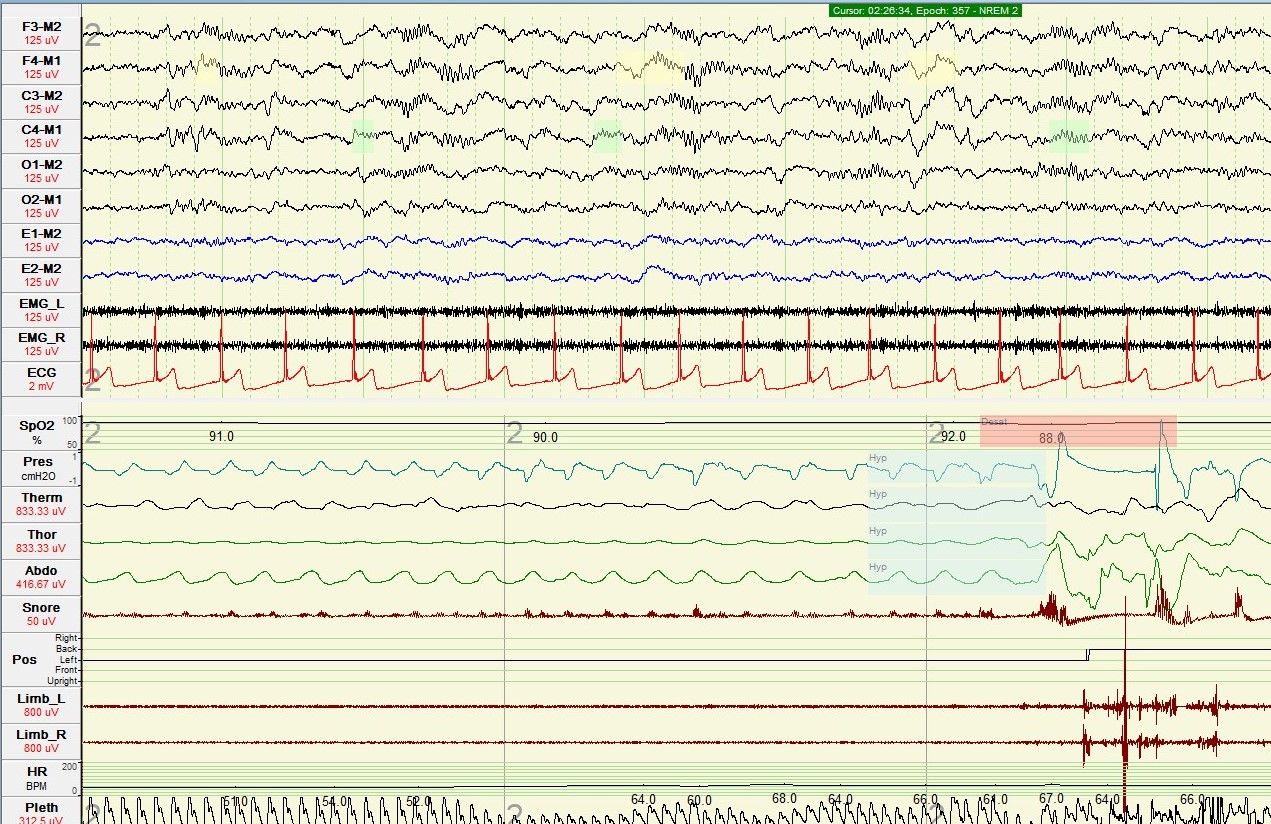

Supplement: Supplementary file 3 [file Image_2.jpg]

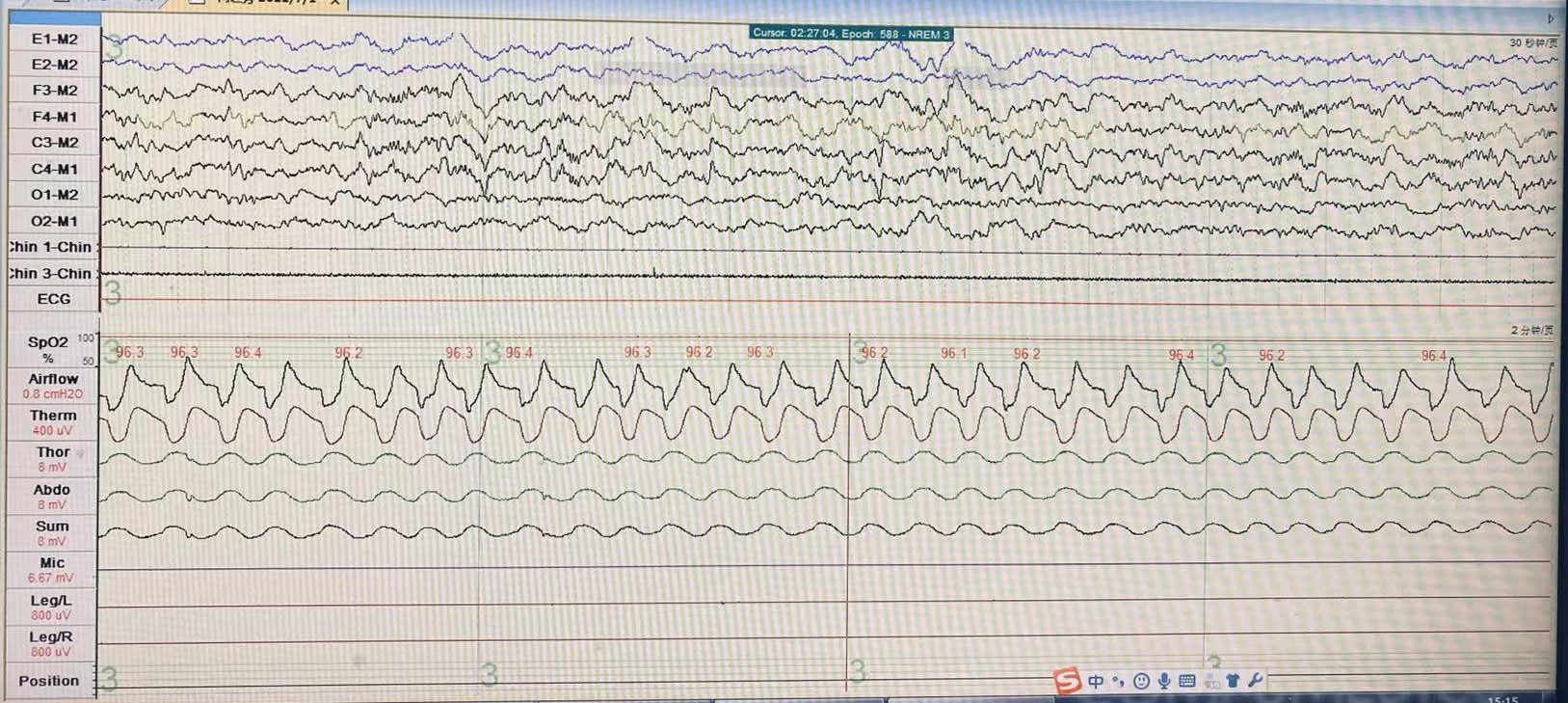

Supplement: Supplementary file 4 [file Image_3.jpg]
